# Evaluation of the Open-Ended Green Chemistry Generic Comparison (GC)^2^ Prompt for Probing Student Conceptions about the Greenness of a Chemical Reaction

**DOI:** 10.1021/acs.jchemed.4c00258

**Published:** 2024-06-27

**Authors:** Krystal Grieger, Alexey Leontyev

**Affiliations:** Department of Chemistry and Biochemistry, North Dakota State University, Fargo, North Dakota 58102, United States

**Keywords:** Second-Year Undergraduate, Chemical Education Research, Organic Chemistry, Testing/Assessment, Green Chemistry

## Abstract

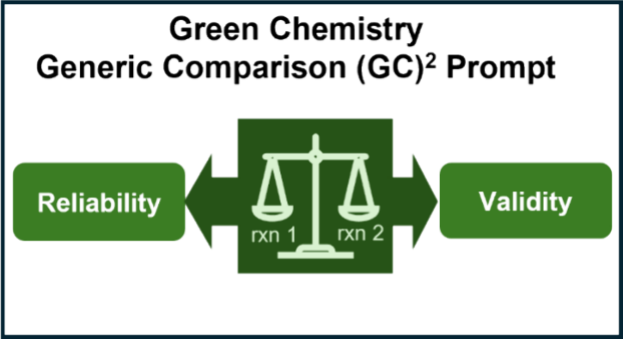

One key limitation for assessing student knowledge of green chemistry is the lack of readily available assessments capable of eliciting reliable and valid data. Therefore, in this study we sought to evaluate the ability of a previously reported open-ended prompt [J. Chem. Educ.2019, 96 ( (11), ), 2410−2419. DOI: 10.1021/acs.jchemed.9b00277] to probe student conceptions about the greenness of chemical reactions. Responses to this prompt, which we titled the *Green Chemistry Generic Comparison* (GC)^2^ prompt, were collected from students enrolled in organic chemistry I and II lecture and laboratory courses (*N* = 642) and from students enrolled in general chemistry II lecture courses (*N* = 272). This prompt was found to be sensitive for detecting gains in green chemistry knowledge in pre- and post- conditions. Furthermore, psychometric analysis revealed that, while addressing certain green chemistry principles was well within the students’ ability range, other principles exceeded that range. These findings indicate that this prompt provides a rapid and effective method for both measuring student knowledge and eliciting student conceptions of the green chemistry principles.

The integration of green and sustainable chemistry (GSC) into the chemistry curriculum has recently moved to the forefront within chemistry education. This emphasis has been echoed globally with the fourth United Nations Sustainable Development Goal stating that by 2030 “all learners acquire the knowledge and skills needed to promote sustainable development.”^1^ In addition, the 2023 American Chemical Society’s (ACS) guidelines for undergraduate chemistry programs now require that ACS certified university curricula provide students with a “working knowledge” of the green chemistry principles (GCPs).^2^ Within these ACS guidelines, *Normal Expectations* now include the incorporation of case studies to demonstrate “the interplay of chemical, environmental, health, regulatory, and business considerations that dictate chemical processes and product design” (p. 9).^1^ Furthermore, as *Markers of Excellence*, students should receive opportunities to assess both chemical products and processes, design greener alternatives when appropriate, and “understand and evaluate the environmental, social, and health impacts of a chemical product over its entire life cycle” (p. 10).^2^ With this focus on green chemistry education (GCE), numerous methods of teaching green chemistry (GC) have been reported, with several recent literature reviews outlining a synthesis of these implementations.^3−9^

In fact, as of 2020, there were over 500 literature articles addressing GSC education, with an increasing number of papers published on the matter each year.^9^ A recent review by Marques et al.^3^ explored the nature of the GCE papers within the *Journal of Chemical Education*, which has published over half of the GCE papers.^4^ They reported that over half of the instructional activities (53%) were implemented within organic chemistry and of those, almost all were integrated into organic chemistry laboratories.^3^ Furthermore, Savec and Mlinarec (2021) found that within the GC perspectives published between 1995 and 2020, the top five taught GCPs were as follows: 1) *using safer solvents and auxiliaries* (GCP#5); 2) *preventing waste* (GCP#1); 3) *catalysis (versus stoichiometric)* (GCP#9); 4) *designing for energy efficiency* (GCP#6); and 5) *use of renewable feedstocks* (GCP#7).^5^ Unfortunately, most of these reported teaching experiences and intervention proposals described only occasional, targeted curriculum insertions and their often poorly evaluated curricular outcomes.^4^ This lack of properly evaluated curriculum can be partially attributed to the matching lack of readily available assessments capable of eliciting valid and reliable data about GC knowledge, which in turn, weakens our overall understanding about the effectiveness of the reported curriculum implementations.

## Green Chemistry Assessment Instruments

Meaningful curricular reform, such as the integration of GCE, requires that curriculum and assessment development happen symbiotically, with a variety of assessment methods available for measuring the impact of the curricular reform.^10^ However, despite the adaptation of the Anchoring Concepts Content Map to include GC concepts^11^ and the development and report of numerous GC curriculum insertions, there remains a limited number of readily available assessment instruments to measure student GC knowledge. Even fewer of these assessments have been evaluated for their ability to provide reliable and valid data. One instrument for which the evaluation of its content validity and psychometric properties was reported was a 13-item two-tier instrument, consisting of both a knowledge and reasoning tier, that was developed to assess Chinese high school student GC knowledge.^12^ Using this instrument, the authors found that high school student understanding of GC concepts increased significantly when comparing scores from students across grade levels with no significant difference in scores between males and females.^12^

In addition, Armstrong et al. (2024) recently developed and published two open-ended GC prompts.^13^ These two prompts evaluated students’ ability to do the following: 1) define GC and 2) compare the preparation of cinnamaldehyde from fossil fuel-derived benzaldehyde versus from steam-distillation of cinnamon tree bark. These two prompts were useful for both eliciting student GC conceptions and measuring GC learning gains due to a GC-enriched general chemistry laboratory curriculum.^13,14^ The second of these prompts is an example of a case comparison between two specific processes that required students know some details about each of the processes.

Similarly, case comparison prompts^15−17^ featuring specific reactions have also been developed and utilized to evaluate student GC learning gains. However, because specific case comparison prompts require that students possess knowledge about the reaction or process to fully answer, these prompts if used as pretests are limited in their ability to elicit all student conceptions about GC since the students may be unaware as to how the GC concept could relate to the problem. Therefore, there is a need to develop prompts that are capable of eliciting student GC conceptions which do not require students to possess specific chemistry content knowledge to answer.

Moreover, we recently developed a 24-item true-false assessment, the *Assessment of Student Knowledge of the Green Chemistry Principles (ASK-GCP)*, to measure undergraduate students’ knowledge of the 12 GCPs.^18^ To assess the utility of this instrument, we collected and analyzed evidence for the reliability of the data and the validity of the inferences that could be drawn from it when used with undergraduate organic chemistry students. Furthermore, we found it to be sensitive for detecting learning gains from multiple interventions, indicating its utility as a pre- and post-test. Finally, while originally designed for undergraduate students, a shortened adapted form of this ASK-GCP instrument was recently employed to measure the learning gains among Brazilian high school students after they completed a case study that dealt with socio-scientific issues concerning water scarcity.^19^

While in the above-mentioned studies this instrument was useful for measuring intervention learning gains and was easy and quick to implement and evaluate, it was also limited because its close-ended format could not uncover student GC conceptions beyond the scope of its specific statements. Furthermore, due to the nature of its statements, the instrument assessed student lower-order cognitive skills.^20^ Thus, in response to these limitations, in this study we sought to explore the functioning of an open-ended prompt from the literature^14^ that asked students to identify the factors they would consider when deciding which of two reactions is greener. It was our hope that this open-ended prompt would assess student higher-order cognitive skills (HOCS)^21^ and uncover both the correct and incorrect GC conceptions and resources that students utilized to construct their responses.

## Open-Ended Assessment Tools in Chemistry

Assessments are used to both convey what information is important and elicit evidence of what students know and can do.^22^ There are a variety of assessment types, such as selected response, constructed response, performance tasks, and portfolios; each type has unique strengths and weaknesses for eliciting student knowledge. Thus, instructors need to use a mixture of assessment types to fully assess student knowledge.^23^ Among these, open-ended assessments are particularly useful for eliciting student conceptions because they require that students apply their knowledge to the problem and thus allow instructors to see which resources students utilize to construct their responses. Due to the open-ended prompts’ ability to elicit student conceptions and reasoning, they have been used for a variety of purposes within chemistry education, including assessing student process skills,^24^ evaluating how course assessments impact what knowledge elements students enlist in their responses about alkene addition reactions,^22^ assessing student knowledge about the particulate nature of matter,^25,26^ and investigating student reasoning about acid–base reactions.^27^ However, despite their utility in eliciting student conceptions for both assessment and research purposes, there is a lack of open-ended assessments focused on GC knowledge.

One type of open-ended assessment that may be well-suited for measuring student knowledge and eliciting student GC conceptions is case comparison prompts.^28^ As noted above, in the ACS Guidelines for Undergraduate Programs, one of the *Markers of Excellence* is that “students are given the opportunity to assess chemical products and processes and design greener alternatives when appropriate” (p. 10).^2^ Thus, by using case comparison prompts students can begin assessing chemical processes and gain an understanding of what features would make a process greener. Furthermore, case comparison activities have been shown to lead to greater learning outcomes than traditional instruction because they prompt students to focus on comparing case features, thus allowing students to generate a schema for the concept that can be applied to new situations.^29^ For further details on the theoretical underpinning and advantages of using case comparisons, refer to the recent work of Graulich and Lieber.^30^

**Figure 1:**
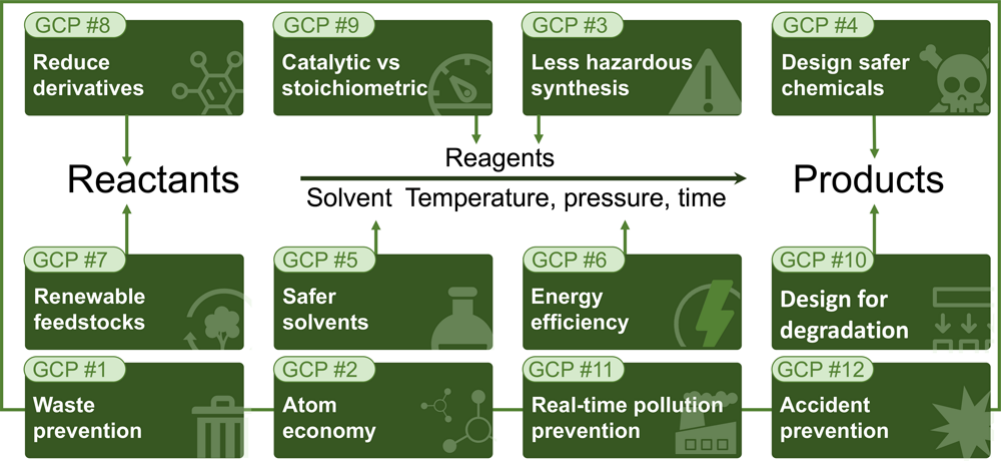
GCPs aligned with starting materials (GCP#7 and 8), solvents (GCP#5), reaction conditions (GCP#3, 6 and 9), products (GCP#4 and 10), and whole reaction (GCP#1, 2, 11 and 12).

Within chemistry, chemical reaction case comparison prompts allow students to mirror the actual inquiry process of organic chemists who often use competitive experiments to uncover reaction mechanisms, allowing students to gain a deeper understanding of the defining features of the reaction and subsequent mechanism.^28^ Similarly, as shown in Figure 1, chemists must also consider the greenness of their chosen reaction materials and conditions and identify whether greener alternatives exist based on the 12 GCPs.^31^ The incorporation of GC case comparison prompts into the curriculum may allow students to gain both a better understanding of this process and of a reaction’s impact on the various climate, ocean, land, and human systems.^32^

## Purpose

In this study, we set out to address the need for an established, readily available, open-ended assessment to measure student GC knowledge that instructors can use to both assess student knowledge and measure the impact of instructional interventions. Thus, we sought to evaluate the validity and reliability of the data obtained from the open-ended *Green Chemistry Generic Comparison* (GC)^2^ prompt, shown in Box 1, that was previously reported by Armstrong et al.^14^

Box 1 **Open-ended *Green Chemistry Generic Comparison* (GC)^2^ prompt reported by Armstrong et al.^14^ that was evaluated in this study**

Suppose there are two reactions that each produce a particular product you desire and that you must choose which reaction is the “greener” reaction. What factors might you take into consideration about each reaction to make this selection? Please list as many as you can.

Please answer with complete sentences that describe the information that is important for your decision. For example, do not just answer “reactants,” but explain what about the reactants you need to know. Note that **the desired product is the same for both reactions** so it should not be a factor that you consider.

There are five textboxes available for you to use. Please put each factor you would consider in a separate box. It is okay if you do not use all five.

While the prompt was provided in the original publication’s Supporting Information as part of the full assessment that students completed, this prompt was neither addressed in the paper^14^ nor any of the follow-up studies.^13^ However, we were particularly interested in this prompt because answering it does not require that students know specific details about the reaction conditions or reagents, thus separating their GC knowledge from their chemistry content knowledge. Moreover, this prompt was potentially suitable for use as a pre- and post-test for intervention studies because it does not require specific chemistry knowledge, which should enable the measurement of learning gains in GC. Due to these useful features, we previously used this prompt as one method for measuring student learning gains when evaluating the effectiveness of our developed GC instructional modules.^16,33^

**Figure 2:**
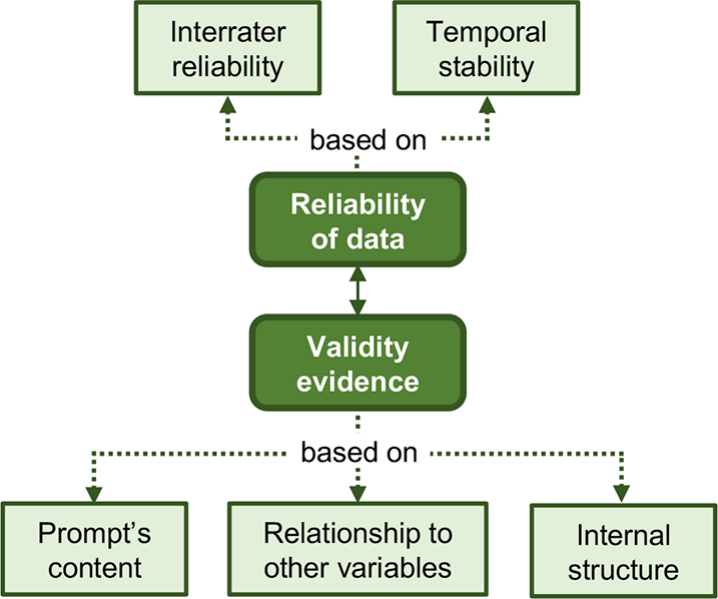
Framework used in the evaluation of the (GC)^2^ prompt.

One key limitation of most reported instruments within not only chemistry, but all discipline-based education research, is that they do not establish the reliability and validity of the data that they collect, which limits the conclusions that can be drawn from them.^34,35^ Therefore, we sought to provide evidence for the validity and reliability of this prompt’s data by evaluating it using the framework (see Figure 2) outlined by the American Educational Research Association (AERA), the American Psychological Association (APA), and the National Council on Measurement in Education (NCME) *Standards for Educational and Psychological Testing*.^36^

## Participants, Setting, and Data Collection

This study was conducted at a medium-sized R1 midwestern university between 2020 and 2023, with the approval of the university’s Institutional Review Board (Protocols IRB0003946, IRB0004053, IRB0004540, and IRB0004714). At this university, the student population consisted of an approximately 49:51 male to female ratio and was predominantly white (∼80%). Furthermore, approximately 40% of first-year first-time students came from rural backgrounds.

Responses to this prompt (*N* = 642) were collected from students enrolled in the following organic chemistry courses: organic chemistry I (Fall 2021, Spring 2022, and Fall 2022), organic chemistry I laboratory (Fall 2020, Fall 2021, and Fall 2022), and organic chemistry II laboratory (Spring 2021). The prompt was administered electronically via Qualtrics as part of student pre- and postassessments for various GC interventions in the lecture and laboratory with students receiving nominal bonus points for completing the assessment. Furthermore, it was administered as a bonus problem within an in-person written lecture exam in both Spring 2022 and Fall 2022 with students receiving 1 bonus point for each correct listed GCP, which corresponded to 1% of the exam total for each correct response.

**Figure 3:**
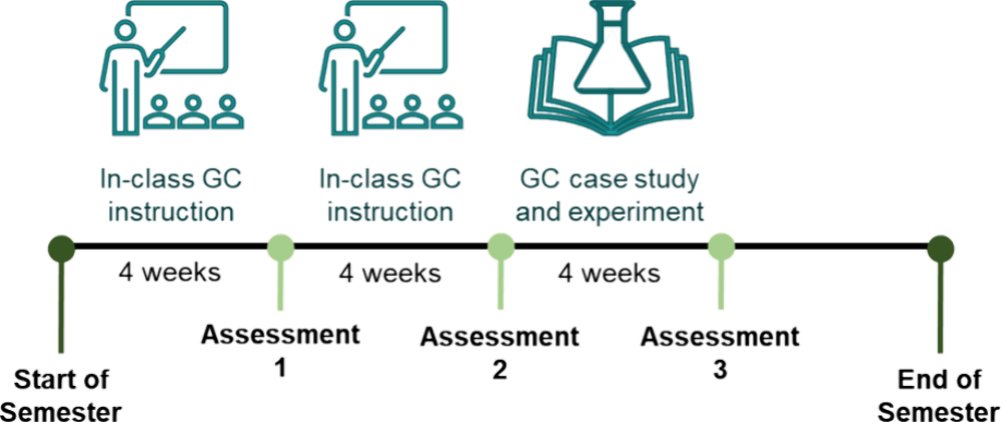
Timeline for the collection of data in the Fall 2022 organic chemistry course.

In Fall 2022, data from students enrolled in organic chemistry I and its corresponding laboratory were collected three times to both monitor student GC learning gains throughout the semester and evaluate the sensitivity of the instrument to measure these learning gains. As shown in the following data collection timeline (Figure 3), students received some GC instruction in lecture prior to completing the first assessment. Students then received further GC instruction in lecture, prior to receiving the second assessment. Finally, students simultaneously completed the GC case study^15^ in lecture and an inquiry-based oxidation experiment in the laboratory in which they compared two methods of oxidizing 5-hydroxymethylfurfural before completing Assessment 3. Data was then analyzed for students who completed all three assessments: Assessment 1 in the lecture and Assessments 2 and 3 in the corresponding laboratories.

In addition to collecting data from the above-mentioned organic chemistry students, data was also collected from students in a general chemistry II lecture course via Qualtrics in Spring 2023 for comparison purposes (*N* = 272). Finally, faculty feedback about the prompt was collected via a Qualtrics survey sent to 74 organic chemistry faculty from across the United States with 22 responding to the survey (response rate = 30%).

## Data Analysis

Student responses for the (GC)^2^ prompt were initially coded to identify which GCPs were addressed. Responses were assigned 1 point for each unique, correct GCP that the student addressed. If a response incorporated more than one GCP, then students could receive an additional point for each unique GCP addressed. However, if two different responses addressed the same GCP, then only one of the responses received a point. In addition, only responses that clearly addressed a GCP received a point. For example, responses that only addressed the speed of the reaction were not assigned a point because the response did not specify its relevance to GC. On the other hand, responses that addressed how the speed of a reaction affects the energy consumed were awarded a point. Similarly, responses that only addressed the difference in temperatures were not given a point because it was not clear which, if any, GCP was being addressed. In fact, some students who elaborated on the difference in temperature addressed the difference in energy, whereas others tied it to laboratory safety. Finally, responses that addressed only which reaction had the higher yield without tying it to the waste prevention GCP were not awarded a point since obtaining a higher yield when comparing two different reactions does not necessarily correlate with preventing waste.

Descriptive statistics for the response score data included the mean, standard deviation, and range of scores. Furthermore, analysis of the normality of the data distribution was conducted, including kurtosis and skewness. All these analyses were performed using StataIC 16. Finally, a Rasch analysis was performed, and a Wright Map was generated in RStudio using the TAM^37^ and WrightMap^38^ packages.

## Results

### Descriptive Statistics

Descriptive statistics were calculated and are shown in Table 1. The possible range of scores was from 0 to 10 because there were only ten applicable GCPs. Only ten of the 12 GCPs were relevant because the prompt indicated that the product was the same for both reactions; therefore, the following two GCPs were not applicable: *designing safer chemicals* (GCP#4) and *design for degradation* (GCP#10).

**Table 1:** Descriptive Statistics from the Results of the Individual Courses

**Group**	***N***	***M***	***SD***	**Range**
General Chemistry II	272	1.4	1.2	0–5
Organic Chemistry I Assessment 1	125	1.1	1.1	0–4
Organic Chemistry I Assessment 2	269	2.4	1.3	0–6
Organic Chemistry I Assessment 3	218	3.0	1.3	0–7

Although there were 10 applicable GCPs, students were only prompted to identify 5 factors in their responses; thus, most students correctly addressed between 0 and 5 GCPs. The average score for general chemistry II was 1.4, whereas for Assessment 1 in organic chemistry I, it was 1.1. The average for Assessment 2 in organic chemistry I was 2.4, and for Assessment 3 in organic chemistry I it was 3.0. This overall progressive increase in scores was expected because students in general chemistry were not explicitly taught about GC, whereas students in organic chemistry I were taught about GC throughout the course. Students responding to the prompt at the end of general chemistry II did better than students responding to the prompt as a bonus on their first exam (Assessment 1) in organic chemistry I. This may be attributed to a difference in administration because some low scores on Assessment 1 were the result of students trying to tie their responses to concepts learned in the course such as acid strength and intermolecular forces instead of just broadly thinking about what would make one reaction greener than another. Furthermore, Assessment 1 in organic chemistry was administered as a paper-and-pencil in-class proctored exam that had to be fully completed in class, whereas in general chemistry II, the prompt was administered via an unproctored Qualtrics survey online without a time limit, which may have impacted the resulting scores.

**Figure 4:**
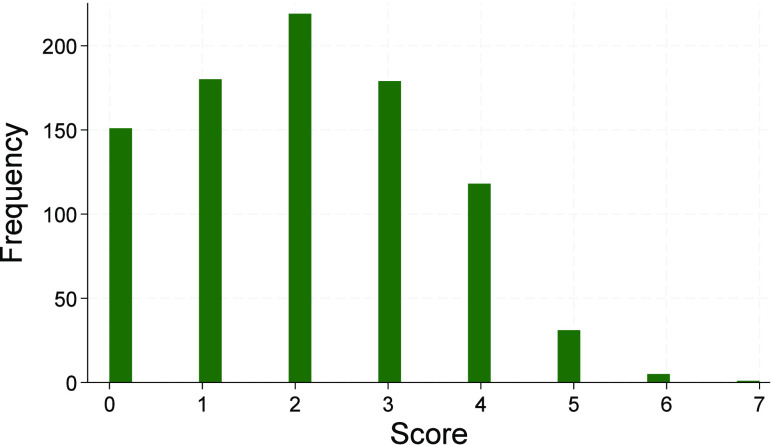
Frequency of student scores obtained from the (GC)^2^ Prompt from GC and OC courses (*N* = 884).

The analysis of the distribution of student scores from all four administrations (Figure 4) indicated that there was neither a floor nor ceiling effect, with few students scoring either a 0 or 5, respectively. Furthermore, the nearly symmetrical distribution indicated that the obtained data followed an approximately normal distribution. This normality of the data was further verified through analyzing its skewness and kurtosis. Skewness refers to any asymmetry in the distribution of data, with a normal distribution of data having a skewness of 0. Analysis of our data indicated a skewness of 0.270, indicating the distribution was slightly skewed to the right. Thus, the mean (M = 2.1) was slightly greater than the median (med = 2.0). Furthermore, kurtosis refers to whether the data is heavy-tailed or light-tailed in relation to a normal distribution, which indicates the frequency of outliers in the data. While a normal distribution would have a kurtosis value of 3,^39^ our data had a kurtosis value of 2.38, indicating it had a lighter-tailed distribution when compared to a normal distribution. However, as shown in Figure 4, the obtained data followed an approximately normal distribution, indicating that the mean and other descriptive statistics were representative of the data.^18,40^

### Reliability

The AERA’s, APA’s, and NCME’s *Standards for Educational and Psychological Testing* defines reliability as the “consistency of the scores across instances of the testing procedure.”^36^ When considered within a traditional chemistry perspective, reliability can be thought of as a measure of an instrument’s precision.

In this study, the reliability of the prompt was measured via interrater reliability (IRR) and test-retest reliability because these aspects are vital when considering the future adoption of an item by other instructors. Since the coefficient alpha is usually reported with close-ended items, it was not an appropriate measure for this case.^41^ IRR was assessed to ensure that the results would be graded consistently among different graders, which is important to consider especially in courses with multiple graders. Furthermore, test-retest reliability was assessed to ensure that the item elicits the same content knowledge from students each time and that other factors do not impact student choice.

### IRR

IRR represents the extent to which two independent raters assign the same score to the same variable.^42^ The most widely used statistic for evaluating IRR is Cohen’s kappa (κ).^42^ In particular, κ is used when two raters rate items using a nominal or categorical scale. It is calculated through comparing the proportion of observed agreements (P_observed_) to the proportion of expected chance agreement (P_chance_) as shown in eq 1.^43^ The resultant values for κ range from −1 to +1, with +1 indicating perfect agreement between raters.^42^
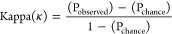
1

Therefore, in this study, Cohen’s kappa was deemed the appropriate statistic to evaluate IRR since both authors independently scored two subsets of student responses based on the presence or absence of each GCP. Comparison of scoring from the first subset of student responses (*n* = 20) resulted in a 55% agreement on overall scores (κ = 0.430). Of those that were scored differently, all but one response had only a 1-point difference in scores. This lower initial agreement is in accordance with other studies whose IRR for open-ended prompts increased in the second round of coding after first round scoring decisions were discussed.^44^ Therefore, in this study, reasons for differences in prompt scoring were first discussed, and then a second subset of student responses (*n* = 10) were independently scored. Comparison of scoring from this subset resulted in a 60% agreement on overall scores with κ = 0.474 indicating a weak agreement between raters.^42,45^ However, all responses that were scored differently exhibited only a 1-point difference. This minor difference in scoring may be mitigated by awarding partial credit for partially correct answers to eliminate the decision of whether to score partially correct responses as either correct or incorrect.

### Test-Retest Reliability

**Figure 5:**
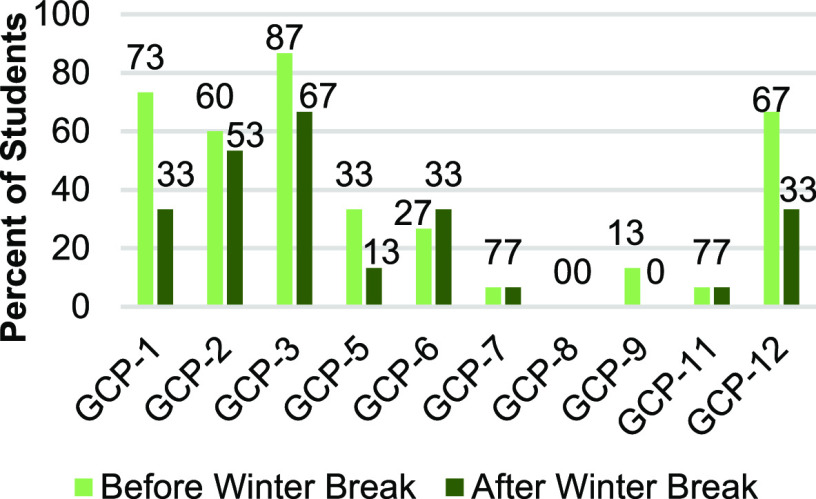
Percent of students mentioning each GCP before and after winter break.

Test-retest reliability refers to the ability of an instrument to elicit the same information from students when taken at two separate time points.^35^ Therefore, the temporal stability of the prompt was measured using data collected from students who were enrolled in both the chemistry majors’ organic chemistry I and II laboratories (*N* = 15) and who responded to the prompt both before (*M* = 3.73, *SD* = 1.22) and after (*M* = 2.47, *SD* = 1.06) winter break. Because students did not receive further instruction about GC over winter break, we expected the prompt’s response scores to remain stable. The average change in an individual’s score after the break was −1.3 with a standard deviation of 0.96, which was calculated by taking the average of the differences between the two administrations. Furthermore, the Pearson’s correlation, *r*(15) = 0.65, *p* = 0.0082, indicated that the scores were strongly correlated, thus providing further evidence for the stability of relative student score ranking. Finally, in addition to the strong correlation in overall scores during both administrations, students provided similar responses with regards to which GCPs were addressed, as shown in Figure 5.

### Validity

The *Standards for Educational and Psychological Testing* defines validity as the “degree to which evidence and theory support the interpretations of test scores for proposed uses of tests.”^36^ Considered within a traditional chemistry perspective, validity can be conceptualized as a measure of the collected data accuracy. The *Standards for Educational and Psychological Testing* states that validity is a unitary concept with various forms of evidence illustrating the different aspects of the instrument’s overall validity.^36^ Within this study, the validity of the inferences that can be drawn from the data collected from the prompt was evaluated through collectively analyzing three main types of validity evidence: 1) evidence based on test content that included using expert judgements; 2) evidence based on the relationship between the assessment and other variables that included the following: convergence of response scores with another established assessment of student GC knowledge, comparison of how groups with different abilities respond, and comparison of how student responses change after they learn about GC; and 3) evidence based on the internal structure of the prompt that included analyzing student responses using Rasch analysis. These types of evidence were selected because they are the most important aspects for instructors interested in using this prompt as either an assessment or as a pre- and post-test instrument.

### Evidence for Validity of the Prompt’s Data Based on the Content of the Prompt

Analyzing the relationship between the test content and the construct to be measured provides strong evidence about the validity of an assessment.^36^ Therefore, to evaluate the evidence for validity based on the prompt’s test content, the prompt was evaluated by organic chemistry instructors who were independent from both the instrument development team and the authors of this study. A survey containing the prompt was sent to 74 organic chemistry faculty from across the United States who had previously^46^ agreed to participate in future studies. A total of 22 faculty responded to the survey resulting in a response rate of 30%. Of the 22 respondents, 5% taught at an institution where the highest degree offered in chemistry was an associate degree, 41% at an institution that offered a baccalaureate degree in chemistry, 23% were at an institution that offered a master’s degree in chemistry, and 36% were at an institution that offered a doctoral degree in chemistry. On average, respondents had taught for 20 years excluding graduate school teaching, with a reported range of 5–43 years of experience.

**Figure 6:**
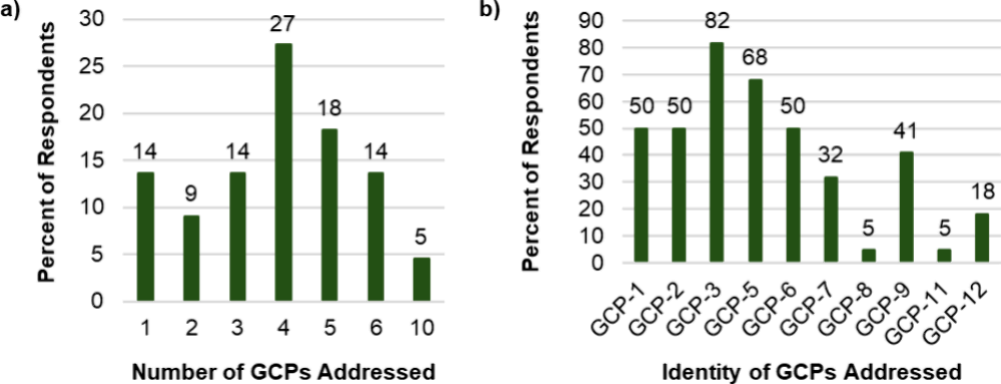
Frequency (a) and identity (b) of GCPs faculty expect their students to provide for the (GC)^2^ prompt.

In the survey, faculty were provided the (GC)^2^ prompt and asked the following question: “After completing your organic chemistry course, what factors would you expect your students to include in their response for this prompt?” As shown in Figure 6, on average, faculty indicated that they would expect students to address 4.0 GCPs (*SD* = 2.1), with a range from 1 to 10 GCPs. Among the responses, the most often mentioned GCPs included *less hazardous synthesis* (GCP#3) and *use of benign solvents* (GCP#5).

Finally, the faculty were asked to provide feedback about the prompt, including word clarity, ability of the prompt to elicit GC knowledge, content importance, and anything else they wished to address. Common feedback included using a comparison between specific reactions instead of the generic prompt to improve clarity (*n* = 4) and providing a list of GC factors for students to consider for their responses (*n* = 2). However, while we are also currently developing and evaluating a series of specific reaction case comparison prompts, the generic nature of this prompt eliminates the need for students to be familiar with the reagents and solvents, making it suitable for use as a pretest. Similarly, while providing the students with a list of factors could be beneficial in some cases since it would give students ideas for what to address, we decided against providing it because we were interested in exploring what conceptions students already held. We therefore chose to evaluate the prompt without incorporating these revisions.

Furthermore, to evaluate the prompt’s word clarity, we calculated the Flesh-Kincaid grade level for the prompt’s wording and found it to be 7.6 with a reading level of eighth to ninth grade.^47^ While, we did not directly evaluate the student response process validity, this lower reading level and the observed student prompt responses indicated that overall students correctly understood what information the prompt was eliciting.

### Evidence for Validity Based on the Relationship between the Assessment and Other Variables

Another aspect that we considered when establishing validity was the evidence based on the relationship between the assessment and other variables. An example of this is convergent evidence,^36^ which refers to how well the instrument relates to other measures of the same construct.^48^ To evaluate the convergent evidence for this prompt, we calculated the Pearson correlation coefficient for the scores that the organic chemistry students obtained on the prompt with the scores that they obtained on the ASK-GCP instrument^49^ when completing Assessments 2 and 3 in the laboratory during Fall 2021 and 2022 (*N* = 487). The Pearson correlation indicated there was a statistically significant, albeit weak,^50^ correlation between the two scores, *r*(487) = 0.28, *p* < 0.0001. This weak correlation is in accordance with findings reported in a literature review of other cognitive assessments in psychology in which there was low evidence (*r* ≈ 0.2) for convergence between close-ended and open-ended assessment items.^51^ In this case, the weak correlation between the (GC)^2^ and the ASK-GCP scores may be attributed to the difference in the cognitive domain levels at which the two instruments assess student knowledge^20^ since the ASK-GCP instrument prompted students to evaluate the correctness of very specific statements about the GCPs, whereas the open-ended prompt focused primarily on a student’s ability to identify and explain what would make a reaction greener. Thus, the ASK-GCP instrument assessed students’ lower-order cognitive skills, whereas the open-ended (GC)^2^ prompt assessed students’ higher-order cognitive skills.

To further evaluate the validity of the obtained responses based on evidence from the prompt’s relationship to other variables, we explored the impact of group differences that were expected to influence student responses to the prompt. Therefore, we applied the known-groups method, which utilizes groups with known differences—in this case students with and without GC instruction. In particular, we gave the instrument to students enrolled in general chemistry II during the last week of the semester (*M* = 1.42, *SD* = 1.20, *N* = 272) who had received little instruction about GC and compared their responses to those from students enrolled in an organic chemistry I laboratory after they received GC instruction through a biobased oxidation experiment in Fall 2021 or through both a green case study^15^ and biobased oxidation experiment in Fall 2022 (*M* = 3.0, *SD* = 1.3, *N* = 218). An independent samples *t*-test was used to compare the difference in scores between the two courses. It was found that there was a significant increase in scores for the organic chemistry I students who received GC instruction, *t*(488) = 14.03, *p* < 0.0001. Furthermore, we were interested in the ability of the prompt to measure the effect size of an instructional intervention. Therefore, we calculated Cohen’s *d* (*d* = 1.280), which is the standardized mean difference between the general and organic chemistry student scores (eq 2). This indicated that on average student scores were higher by 1.3 standard deviations with GC instruction; thus, illustrating the utility of the prompt for measuring the effect size of an intervention.
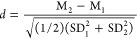
2

Finally, similar to the known-groups paradigm, it was expected that as students learned more about GC, their scores on the assessment would improve. We were particularly interested in evaluating the prompt’s sensitivity for measuring learning gains from multiple instructional interventions. Therefore, the prompt was administered three times during a semester of organic chemistry I in Fall 2022 (as shown in Figure 3). Only data from students enrolled in both the corresponding lecture and laboratory courses, who completed all three assessments, and consented to using their responses in the study were used in this analysis (*N* = 68). Students first responded to the prompt as a bonus question on their first written exam in the lecture (Assessment 1). Approximately one month later, after some in-class instruction on GC, students responded to the prompt again on Assessment 2 which was administered in preparation for an oxidation experiment in the laboratory and a GC case study in the lecture. This prompt was graded for completion only and administered via Qualtrics. The students then simultaneously completed the biobased oxidation experiment in the laboratory and an oxidation–reduction case study featuring GC concepts^15^ in the lecture. Finally, the students responded to the prompt one last time on Assessment 3 via Qualtrics. The average normalized learning gains between Assessment 1 and Assessment 2 and between Assessment 2 and Assessment 3 were calculated by taking the average of the individual learning gains between individual prompt scores.^52^ The individual learning gains (*g*) were calculated using eq 3, and then the average normalized learning gain was calculated by taking the average of all individual learning gains. Due to the nature of the calculation, students who scored a 100 on either Assessment 2 for Gain I or Assessment 3 for Gain II were excluded from the analysis.
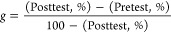
3

The resultant average scores and normalized learning gains from students who completed all three assessments are illustrated in Table 2. As shown, medium learning gains for both interventions were observed, indicating that students continued to benefit from both instructional interventions and that the prompt was effective in measuring these gains.

**Table 2:** Average Scores and Normalized Learning Gains of Students Completing All Three Assessments

	**Assessment**			**Gain**^a^	
	**1**	**2**	**3**	**I**	**II**
*M*	0.99	2.16	3.16	0.61	0.62
SD	1.10	1.17	1.29	0.95	0.98

a Gain I between Assessments 1 and 2 is due to in-class instruction, whereas Gain II between Assessments 2 and 3 is due to students completing a green chemistry case study and biobased oxidation experiment.

**Figure 7:**
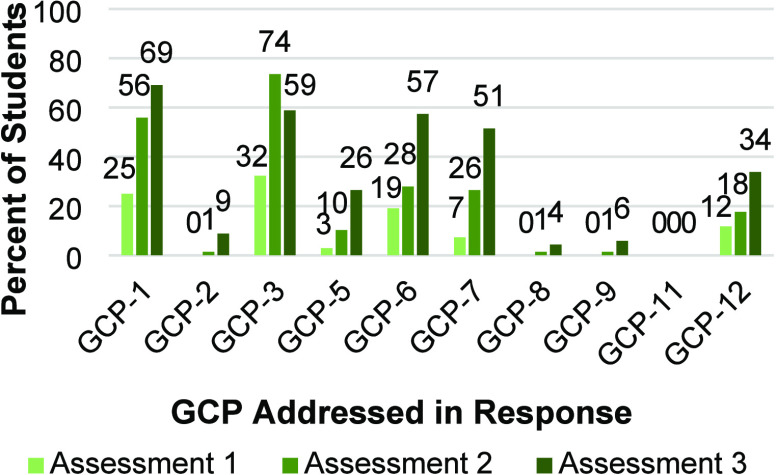
Percent of students addressing each GCP on Assessment 1 to Assessment 3.

To evaluate the ability of the prompt to detect changes in student knowledge between each administration, a one-way repeated measures ANOVA was conducted. A statistically significant difference was observed, *F*(2, 134) = 73.76, *p* < 0.0001; the Sidak posthoc analysis further indicated that there was a significant difference in scores between each administration. Furthermore, the percentage of correct responses for each GCP provided by students on each administration was compared. As shown in Figure 7, the percentage of students correctly addressing each GCP increased throughout the semester, providing further evidence for the prompt’s ability to measure student learning gains from multiple interventions.

### Evidence for Validity Based on the Internal Structure

To evaluate the prompt’s internal structure, we sought to identify whether the actual student difficulty for correctly identifying each GCP corresponded to the expected difficulty. It was expected that the GCPs relating to *waste prevention* (GCP#1), *less hazardous synthesis* (GCP#3), and *design for energy efficiency* (GCP#6) would be easiest for students to answer since these principles relate to environmentally friendly concepts that students are taught throughout their lifetimes. Similarly, we expected students to be less familiar with the concepts of using *catalytic versus stoichiometric quantities* (GCP#9) and *real-time analysis for pollution prevention* (GCP#11) since these concepts are not commonly thought about in daily life or taught in the curriculum. While students are taught about the use of catalysts in organic chemistry, there is typically less emphasis on catalytic loading or its role in waste prevention. Thus, while we expected students to address the use of catalysts, we did not expect them to correctly address how their use contributes to the greenness of a reaction. To identify whether these expected difficulties matched the actual difficulties, we utilized Rasch analysis to analyze the responses from the students in general and organic chemistry.

Rasch analysis is a type of psychometric technique within item response theory that allows a latent trait, such as GC knowledge, to be measured. Rasch analysis allows researchers to measure a respondent’s performance on an instrument via a linear scale that accounts for the items having difficulties that range from easy to hard.^53^ In fact, Rasch analysis makes use of these item difficulty rankings as the marks of a linear scale for measuring the latent trait, in this case student knowledge of the GCPs.

Because it considers the impact due to the disparity in problem difficulty, within Rasch analysis the intervals represented between different raw instrument scores are considered unequal. For example, the difference in knowledge between students who answer 50% vs 55% of the items correctly is smaller than students who answer 95% vs 100% of the items correctly due to the more difficult questions requiring more content knowledge to answer.^54^ In fact, Rasch analysis operates on the principle that the raw scores represent a distribution of knowledge that follows a normal curve.^54^ Thus, a student’s probability of correctly answering a question is dependent only on their ability and the item difficulty. The probability of correctly answering a question, *P*_*ni*_(x = 1), is therefore calculated as a function of the difference between a person’s ability, *B*_*n*_, and the item’s difficulty, *D*_*i*_, which is illustrated in eq 4.^55^ Within this equation, person ability, *B*_*n*_, refers to a respondent’s odds-of-success for a given task, while item difficulty, *D*_*i*_, refers to a respondent’s odds-of-failure for the task.^18,56^
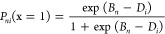
4

The resulting logit scale allows one to predict the probability of correctly answering a question, or in this case correctly addressing one of the GCPs in their responses, based on student ability. When the student ability is equal to item difficulty, the student has a 50% chance of correctly answering the item. Similarly, students would be expected to correctly answer items whose difficulties are less than their ability and would not be expected to correctly answer items whose difficulties are higher than their ability.

Due to the resulting logit scale relating student performance to item difficulty, its use is particularly valuable for analyzing instruments because it allows the results to be generalized to the greater population and not be sample dependent. Stated another way, different samples may vary on their ability and where they fall on the scale, but the determined order of item difficulties remains constant. Furthermore, the Rasch analysis provides researchers with a method to evaluate the structure of an instrument and identify the hierarchy of item difficulty. Thus, through the Rasch analysis of this prompt we identified the hierarchy of difficulty for students to address each of the GCPs in their responses. Due to the useful information that it generates, Rasch analysis has been used in a variety of studies within chemistry education. These include analyzing the quality of instrument^18,57−59^ and assessment^60,61^ items, comparing student performance on in-person versus online exams,^62^ and investigating student understanding of middle school science concepts as they progress from middle school through college.^63^

To conduct Rasch analysis on the student responses from general and organic chemistry, each student response was evaluated to identify which of the 10 applicable GCPs were correctly addressed. Correctly addressed responses for each GCP were coded as 1 and incorrect or not addressed GCPs were coded as 0. Furthermore, Rasch analysis requires that the instrument’s items exhibit unidimensionality, local independence, and monotinicity.^64^ In this case, the data obtained from the (GC)^2^ prompt exhibits unidimensionality because the responses are to a single prompt which was designed to measure student knowledge of the GCPs. Furthermore, local independence refers to the responses being independent from one another with the correctness of one response not influencing the correctness of a different response. Since students were prompted to provide five unique statements and the grading for each response was conducted independently from their other responses, the local independence assumption was met. Finally, monotonicity refers to the correlation between an item score and person ability with a greater person ability expected to yield a higher score.^64^ Since all three conditions were met, Rasch analysis was appropriate for analyzing the data.

**Figure 8:**
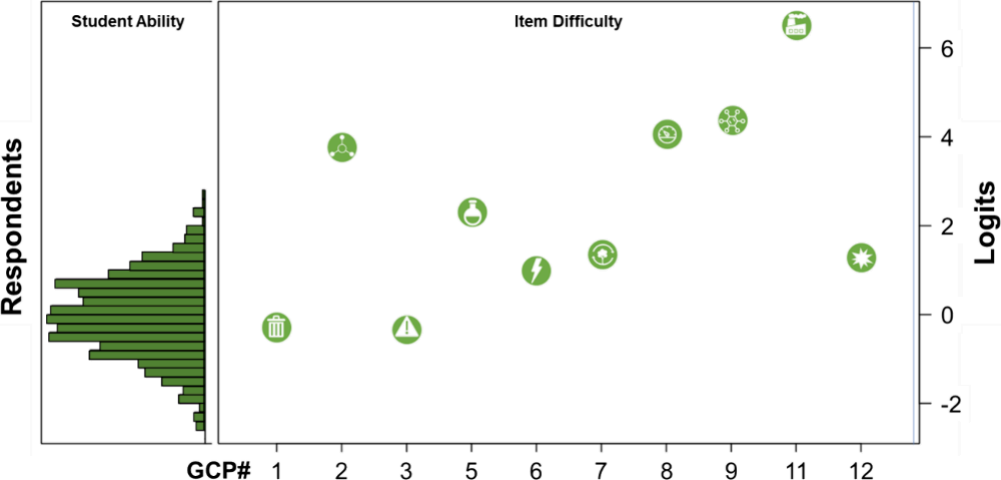
Wright map illustrating distribution of person ability on the right versus item difficulty for each of the GCPs on the left (except the inapplicable GCP#4 and 10) plotted on a logit scale. The more often mentioned GCPs have a lower logit value than less often mentioned GCPs.

Within Rasch analysis, Wright maps are used to illustrate the relationship between student ability and item difficulty. A key feature of Wright maps is that item difficulty uses the same linear scale—logits—as the person measure of student ability.^65^Figure 8 illustrates the Wright map generated for this study. The right side of the map denotes the distribution of student ability, whereas the left side of the map represents the item difficulties for addressing each of the GCPs. As shown in Figure 8, the GCPs that students most often correctly included in their responses were *less hazardous synthesis* (GCP#3), *waste prevention* (GCP#1), and *design for energy efficiency* (GCP#6) with item difficulties of −0.338, –0.292, and 0.945 logits, respectively. These responses were in accordance with the GCPs that we expected would be easier for students to address and also with three of the five GCPs that faculty indicated they would expect their students to be able to address by the end of their organic chemistry course. Furthermore, analysis of the responses from the general and organic students indicated that while correctly addressing certain GCPs was well within the student ability range, other principles exceeded that range. This was especially true for the GCP *real-time analysis for pollution prevention* (GCP#11), for which none of the students correctly addressed the principle in their responses. This correlates with both our prediction and the expectations expressed by other faculty for their students since only one respondent indicated that they would expect their students to be able to address this GCP. The lack of attention for this principle is also illustrated in the literature with only 4% of the GCE papers addressing *real-time analysis for pollution prevention*.^5^ Moreover, due to the focus of this principle, it is more likely to be emphasized within analytical chemistry courses than organic chemistry. However, since most students taking organic chemistry do not proceed to analytical chemistry, which is typically only taken by chemistry majors, there is a need to further explore the difficulties with addressing this principle and to develop curriculum for teaching students about it within organic chemistry.

As shown in Figure 8 and described previously, the difficulties for the GCPs addressed in the student responses matched what we had expected, thus providing evidence for validity based on internal structure. This evidence when taken together with the evidence for validity based on both the content and the relationship between the assessment and other variables supports the validity of the inferences that can be drawn from the data collected using the (GC)^2^ prompt when administered to students in undergraduate organic chemistry.

## Limitations

This prompt has been shown to effectively assess student knowledge of the GCPs; however, as with all instruments, there are limitations which must be acknowledged in order to aid instructors in their adoption of the prompt and interpretation of the data they collect. These limitations include the limited evidence for response process validity, the administration of the instrument at only one institution, and inconsistencies in student scores for brief or partially correct responses.

### Response Process Validity Evidence

Response processes refer to what people do, think, or feel when responding to a task or item and can lead to variation in resulting response scores.^66^ While student responses to the item indicated that they understood what the prompt was asking, we did not thoroughly explore response process validity for this prompt. Therefore, future studies should include cognitive interviews with students completing the instrument based on Tourangeau’s four stage cognitive model of response process.^67^

### Instrument Assessed at Only One Institution

While this prompt has been used in courses taught by different instructors, all analyzed responses were obtained from one R1 institution in the Midwest. Thus, the prompt’s sensitivity for measuring the effect of GC instructional implementations on student learning may vary due to differences in the nature, intensity, or duration of the instructional implementation or due to the population being studied. Therefore, future studies should investigate the utility of the prompt at different institutions and with various GC instructional implementations.

### Inconsistencies in Scoring Brief or Partially Correct Student Responses

Among the responses we received, some statements were too brief, mildly vague, or were only partially correct, which led to discrepancies when the authors scored them. While the brevity of these responses may be due to providing the prompt in instances where it was either used as a pretest before instruction on GC, graded only for completion, or used as a bonus question at the end of an exam, this inconsistency in scoring responses should be considered when using multiple graders for a course. Therefore, we recommend that if using multiple graders, discussions throughout the grading process should include the following: 1) what instructional GC content has been taught in the course and what are the expectations for responses addressing that content; and 2) how to grade the unclear, vague, or very brief responses that occur. Furthermore, a prepared answer key outlining acceptable responses should be provided to the graders and updated throughout the grading process based on the results of the grading discussions.

## Implications for Practice

It is our hope that other chemistry instructors utilize this open-ended *Green Chemistry Generic Comparison* (GC)^2^ prompt, which has been found to be sensitive for measuring changes in GC knowledge from instructional interventions, in their courses to measure their students’ GC knowledge. Because of its generic nature, it can assess student GC knowledge without requiring students to know any details about specific chemicals or reactions, which can be helpful for both pretests and formative assessments. Through identifying student conceptions and incorrect ideas, instructors can identify areas that need further instruction and build from students’ correct conceptions and resources. Furthermore, the incorrect ideas provided by students can be used to generate distractor items for multiple choice questions^68−70^ or be used to generate a *Measure of Linked Concepts* assessment^71^ in which students mark each of a compiled list of student responses as either true or false, thus allowing instructors to see the prevalence of incorrectly applied resources or incorrect conceptions.^72^ Finally, in our administration of the prompt, the elicited student responses typically addressed the same concepts; therefore, future work should explore the utility of machine-scoring student responses.^73−75^

This (GC)^2^ prompt can be administered via either paper-and-pencil assessments or online through survey tools such as Qualtrics, Google Forms, or using the course Learning Management System. We recommend that faculty include designated spaces, either numbers or boxes, after the prompt for each student response because in this study these designated spaces promoted the organization of the responses, which, in turn, facilitated both easier and quicker grading. Finally, we also recommend that instructors generate a key, keeping it updated with new correct student responses to ensure consistent grading across graders.

## Implications for Research

We hope that the methodology used in this study will serve as a framework for other discipline-based education researchers interested in the development and evaluation of open-ended assessments. Furthermore, through exploring student responses to this prompt in conjunction with their responses to other assessments, researchers can develop and explore a nomological network^76^ of interconnections with other cognitive, affective, and behavioral variables predicted to be linked to GC knowledge. This resulting network will allow for a greater understanding of the nuanced aspects of the overall impact that GC instruction has on our students.

In addition to being used to explore the nomological network, student responses to this prompt can also be analyzed as they progress from being a novice to an expert on GC. Used in conjunction with other studies, this analysis could help identify common learning progressions for GC knowledge.^77,78^ Subsequently, this identified learning progression can be used to decide how the GC content should be integrated into each progressive chemistry course to best support student learning of the material.

Finally, having chemistry education researchers identify and use a uniform assessment method for the various reported instructional GC interventions will be essential to compare the relative effectiveness of a proposed novel intervention in relation to existing interventions. Since using a combination of different assessment types has been found to be the most effective for measuring an intervention’s impact,^23^ we recommend using this prompt in conjunction with other instruments that assess both the cognitive domain, such as the defining green chemistry prompt,^13^ the ASK-GCP instrument^18^ and specific reaction case-comparison prompts,^13,15−17^ and the affective domain, such as the *Attitude toward Subject of Chemistry Inventory*,^79,80^*Academic Motivation Scale-Chemistry*,^81^ and the *Achievement Emotions Questionnaire-Organic Chemistry*.^82^ A review outlining a compendium of literature examples of assessment methods that can be used to assess student learning and attitude outcomes of GC instruction can be found elsewhere.^83^ Furthermore, for a broader range of assessment tools in chemistry, the Chemistry Instrument Review and Assessment Library (CHIRAL) contains information about over 500 assessment tools, where users can easily find instruments related to a specific domain, topic, or format.^84^

## Conclusions

In conclusion, this study set out to evaluate the ability of an open-ended prompt, the (GC)^2^, to probe student conceptions about the greenness of a chemical reaction. The prompt was found to be suitable as an assessment measure for pre/post research design due to its sensitivity in detecting learning gains in GC knowledge. In addition, due to its open-ended nature, the prompt was capable of eliciting student conceptions about GC. Finally, a psychometric analysis of the responses indicated that while addressing certain GCPs, such as *waste prevention* (GCP#1), *less hazardous synthesis*, (GCP#3), and *design for energy efficiency* (GCP#6) were well within student ability range, other principles, such as *real-time analysis for pollution prevention* (GCP#11) exceeded that range. This indicates the need to develop and implement curricula that specifically address the concepts students struggle with using the framework outlined by Day et al.^85^ Specifically, the identified difficulty order of the green chemistry principles can inform the implementation of their Design Principle #2 which suggests that the complexity of sustainability issues in the curriculum should increase over time.^85^ It is our hope that this study’s findings support the adoption of this prompt among GC educators as they measure the impact of the GC curriculum on their students’ GC knowledge and conceptions.
